# Assessment of Fatty Acid Profiles and Proximate Composition of 10 Cambodian Farmed Freshwater Fish

**DOI:** 10.1155/ijfo/5927997

**Published:** 2026-05-26

**Authors:** Sovannmony Lay, Chanvorleak Phat, Hasika Mith, Vattana Mom, Cédric Delforge, Sylvie Gobert, Samiha Boutaleb, Marie-Louise Scippo, Caroline Douny

**Affiliations:** ^1^ Research and Innovation Center, Institute of Technology of Cambodia, Phnom Penh, Cambodia; ^2^ Faculty of Chemical and Food Engineering, Institute of Technology of Cambodia, Phnom Penh, Cambodia; ^3^ Laboratory of Food Analysis, FARAH-Veterinary Public Health, University of Liège, Liège, Belgium, ulg.ac.be; ^4^ Laboratoire d′Océanologie, Centre MARE, Université de Liège, Sart Tilman, Liège, Belgium, ulg.ac.be

**Keywords:** DHA, EPA, farmed fish, fatty acids, minerals, nutritional contribution, nutritional quality

## Abstract

Globally, aquaculture production has continued to rise, surpassing capture fisheries production for the first time, underscoring its pivotal role in meeting the growing global demand for aquatic foods. This growth emphasizes its significance in addressing food security and offering high‐quality protein and necessary nutrients to growing population. Fish in Cambodia is a nutritional primary source, accounting for 75% of total animal protein intake, making it an indispensable part of national food security and nutrition initiatives. However, the threat of many factors, such as the increase of hydropower dams, water pollution, and illegal fishing, led to the reduction of inland fisheries. Consequently, aquaculture systems have rapidly emerged as an alternative source of food, nutrition, and livelihood. Therefore, this study aimed to evaluate the nutritional quality of commercialized farmed freshwater fish to provide more insight regarding nutritional information and scientific data. Ten different farmed freshwater fish species were analyzed for proximate composition, fatty acid profile, essential dietary elements, nutritional quality indices, and nutritional contribution to human health. The results revealed that moisture content varied from 63.37 ± 0.37 g/100 g to 79.98 ± 0.72 g/100 g, lipid content ranged from 1.60 ± 0.1 to 17.82 ± 0.04 g/100 g fish, protein content ranged from 16.08 to 21.91 g/100 g fish, and ash content varied from 0.51 ± 0.04 g/100 g to 2.49 ± 0.31 g/100 g. Saturated fatty acids were found at the highest value (40.4–56.3 g/100 g of total fatty acids), followed by polyunsaturated fatty acids (26.4–51.5 g/100 g of total fatty acids) and monounsaturated fatty acids (3.5–29.9 g/100 g of total fatty acids). In addition, the PUFA/SFA ratio varied from 0.5 to 1.34, and the ratio of n‐6/n‐3 ranged from 0.6 to 11.1. Furthermore, the AI and TI index values were calculated at values ranging from 0.5 to 1.13 and 0.39 to 1.24, respectively. The value of the h/H index in farmed fish species ranged from 0.63 to 1.76. The studied farmed fish species provide high content of calcium (43.6–8612 mg), potassium (1581.9–3723.2 mg), and phosphorus (1019.9–5890.1 mg). Moreover, a 90‐g daily serving of walking catfish provides 9400 mg of EPA + DHA, covering 169% of the daily recommended intake for adults. The findings provided baseline data of fatty acid profiles for nutritional assessment, informed public health strategies, and supported the sustainable development of the aquaculture sector.

## 1. Introduction

Currently, aquatic resources have become increasingly important for food security and nutrition. Nevertheless, further efforts are required to meet the demands of a rapidly urbanizing population [1]. In 2022, global aquatic animal production was estimated at 185.4 million tons. At the same time, the production of aquaculture surpassed a record high of 130.9 million tons, including 94.4 million tons of aquatic animals.

Simultaneously, fish play a crucial role in Cambodia, with the nation heavily depending on its aquatic resources and biodiversity to ensure food security and sustain livelihoods [2]. Cambodia exhibits one of the highest documented per capita consumptions of freshwater fish globally (33 kg/capita/year) [3, 4]. Moreover, in Cambodia, fish provides 76% of household protein intake and is the second most popular food after rice [5]. Recently, research and development institutions have only begun to pay significant attention to aquaculture. This interest aligns with the anticipated and now increasingly evident drop in the production of inland capture fisheries. For instance, the Tonle Sap′s fish catch reportedly declined by 23% in 2020 due to a combination of drought and water impoundment from upstream dams, sparking concerns over an impending fisheries collapse [2]. This observation coincides with the presence of a highly biodiverse freshwater fish fauna, encompassing over 400 species. In general, inland fisheries account for the majority of fish consumption and supply to local and international markets, with marine fisheries accounting for a small percentage; however, the collapse in inland fisheries′ fish stocks prompted the aquaculture industry to contribute to rural livelihoods [2]. The major cultured species are *Pangasius* sp. (73%), followed by giant snakehead (*Channa micropeltes*) (21%). Other species produced include *Puntius* sp., Philippine catfish (*Clarias batrachus*), marble goby (*Oxyeleotris marmorata*), *Cirrhinus* sp., red‐tailed tinfoil (*Barbonymus altus*), and Hoven′s carp (*Leptobarbus hoeveni*) [3, 4].

Fish is traditionally regarded as an affordable protein source that benefits people worldwide and has significant nutritional value [6]. Because of its high‐quality protein, well‐balanced amino acids, low saturated fat content, and high omega‐3 fatty acid content, as well as its vital microcomponents such as vitamins and minerals to support consumers′ overall health, fish is regarded as one of the most nutrient‐rich animal‐derived products [7–9]. Fish protein is easily absorbed, and its amino acids support a wide range of physiological functions, such as tissue repair, equilibrium, control over several cellular functions, function as precursors to hormones and nitrogenous bases, and support healthy growth in individuals of all ages [10].

In addition to being an important source of protein, freshwater fish also contain lipids and semi‐essential fatty acids, which are beneficial for human health [11, 12]. The phosphoglycerides of cellular membranes in fish contain a high concentration of omega‐3 polyunsaturated fatty acids (PUFAs), particularly eicosapentaenoic acid (EPA; 20:5 n‐3) and docosahexaenoic acid (DHA; 22:6 n‐3). Among these n‐3 LC‐PUFAs, DHA is physiologically important in neural tissues. DHA is prevalent in the retina and brain, where it plays a vital role in preserving the structure and functionality of the excitable membranes in these tissues [11]. Moreover, EPA is the precursor of the extremely physiologically active class of chemicals known as eicosanoids, which have a variety of physiological activities, including blood coagulation, the immune system, inflammation, cardiovascular tone, renal and neurological function, and reproduction [11]. For n‐6 fatty acids, because there was no evident benefit of n‐6 PUFAs on cardiovascular protection, nutritionists have suggested increasing n‐3 and decreasing n‐6 intake. However, n‐3 fatty acid intake remains low in many nations′ human populations, whereas n‐6 fatty acid consumption from vegetable oil and animal fats continues to rise [13]. A diet that falls between 1/1 and 1/2 for the n‐3/n‐6 PUFA ratio is said to be well‐balanced in fatty acids. However, research indicates that the PUFA ratio in the modern Western diet is unbalanced, ranging from 1:15 to 1:25 (n‐3/n‐6), which has been associated with an increased risk of chronic inflammatory conditions, cardiovascular diseases, and metabolic disorders [12].

Although fish are widely recognized for their health‐promoting properties, their nutritional and biochemical profiles vary considerably both across and within species. These variations are primarily influenced by multiple intrinsic and extrinsic factors. Intrinsic factors are, for instance, biological characteristics inherent to the organism, such as taxonomic position and sex, significantly influencing lipid metabolism and deposition of omega‐3 long‐chain PUFAs (EPA and DHA) [14]. Extrinsic factors such as feeding behavior, seasonal variation, environmental (biotic and abiotic) conditions, including water temperature, pH, and the quantity and type of available feed, contribute to differences in total fat content and fatty acid composition [7, 15]. For instance, various seasons affect the lipid content in fish, which may vary from 4% to over 30% in mackerel and 2%–25% in herring [16]. It has also been observed that larger fish carry a higher concentration of saturated fatty acids (SFAs) compared to those of medium and small‐sized fish [16].

With the rising contribution of farmed fish to global fish supplies, ensuring the preservation of their high‐quality lipid profile, particularly the content of beneficial omega‐3 PUFAs, becomes increasingly critical. This is particularly relevant in Cambodia, where a study by Sroy et al. [17] suggests a potentially low PUFA content in commonly consumed wild freshwater fish, and there is a lack of information on the nutritional composition of farmed freshwater fish in Cambodia. This knowledge gap limits the capacity to provide evidence‐based dietary recommendations and enhance aquaculture practices for improving nutritional outcomes. Therefore, the objective of this research was to characterize the proximate composition, element compositions, fatty acid profiles, nutritional quality indices (NQI), and nutritional contribution in 10 commonly farmed freshwater fish species in Cambodia. A preprint of this work has previously been posted [18].

## 2. Materials and Methods

### 2.1. Sample Collection and Preparation

**Figure 1:**
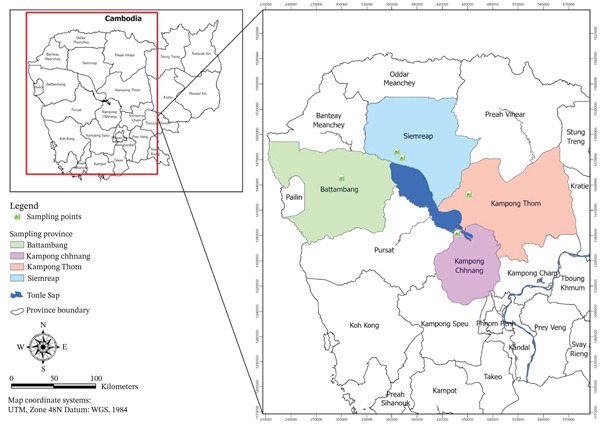
Geographical location of sampling points of Cambodian farmed freshwater fish species. *Note: this figure was created by authors.*

**Figure 2:**
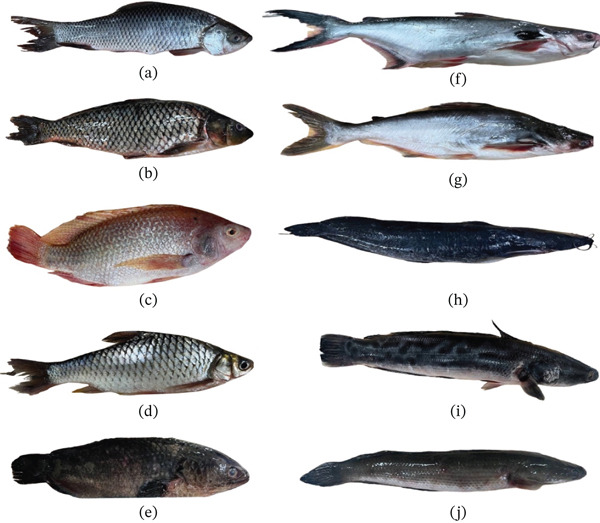
Pictures of Cambodian farmed freshwater fish species used in this study: (a) *Labeo rohita* (IC), (b) *Cyprinus carpio* (CC), (c) *Oreochromis niloticus* (NT), (d) *Barbonymus gonionotus* (SB), (e) *Anabas testudineus* (CP), (f) *Pangasius larnaudii* (BEC), (g) *Pangasius hypophthalmus* (PG), (h) *Clarias batrachus* (WC), (i) *Channa micropeltes* (GSH), and (j) *Channa striata* (SSH).

Fish were purchased from four farmers located in four provinces in Cambodia, including Kampong Thom, Siem Reap, Battambang, and Kampong Chhnang (Figure 1). Ten farmed freshwater fish species, which are commonly cultured and consumed by Cambodian people were identified through a previous survey based on interviews and conducted in April 2023 across major aquaculture‐producing regions of Cambodia (unpublished data), ensuring representation based on production prevalence and market availability. Those included *Channa striata*, *Barbonymus gonionotus*, *Cyprinus carpio*, *Labeo rohita*, *Pangasius hypophthalmus*, *Anabas testudineus*, *Pangasius larnaudii*, *Oreochromis niloticus*, *C. micropeltes*, and *C. batrachus* (Figure 2). Farmers used various foods to feed the fish (pumpkin, Moina, industrial feed…), depending on their availability during the growing season.

For each sampling site, approximately 2–3 kg of each farmed fish species was collected, except for *L. rohita* (*N* = 1; 572 g) due to limited availability during the sampling period. The number of fish collected to obtain 3 kg is mentioned in Table 1. After being harvested, since the fish was not beheaded and eviscerated by the farmer, the fish were immediately packed in a plastic bag and stored under ice (0°C) in a polystyrene box to prevent perishability and maintain their nutritional quality. After that, they were transferred to the laboratory within 1 h for storage at −20°C for further analysis. Each fish species was identified, and weight, length, and width were individually measured for all fish. Fish samples were then prepared by removing organs (scales, fins, and gills) and filleted with skin. Then, the fillets from each species were recombined and cleaned with distilled water before being ground using a grinding machine in a room where the temperature was 20°C ± 2°C to minimize fatty acid oxidation. Sample preparation was done within 1 h. Part of the mixed sample was used to determine the moisture and ash content, whereas the other part was freeze‐dried for the determination of other nutritional parameters.

**Table 1:** Morphometric data and proximate composition of farmed freshwater fish species.

Species	Weight [g]	Length [cm]	Width [cm]	Moisture [g/100 g wet weight] (*n* = 3)	Ash [g/100 g wet weight] (*n* = 3)	Lipid [g/100 g wet weight] (*n* = 3)	Protein [g/100 g wet weight] (*n* = 3)
*C. striata* (*N* = 3)	751.3 ± 24.5	40.3 ± 1.1	7.5 ± 0.1	78.1 ± 0.6	0.77 ± 0.12	3.69 ± 0.4	16.8 ± 0.64
*B. gonionotus* (*N* = 7)	306.1 ± 72.9	25.5 ± 3.6	8.2 ± 1.5	74.2 ± 0.6	0.59 ± 0.01	8.35 ± 0.1	16.3 ± 0.62
*C. carpio* (*N* = 6)	270.0 ± 58.7	26.0 ± 3.6	7.6 ± 1.0	74.4 ± 1.4	0.52 ± 0.06	3.27 ± 0.19	21.2 ± 0.81
*L. rohita* (*N* = 1)	572	37.35	8.65	73.6 ± 0.01	1.29 ± 0.02	1.60 ± 0.1	21.9 ± 0.83
*P. hypophthalmus* (*N* = 3)	752.6 ± 20.7	45.6 ± 1.3	8.8 ± 0.4	71.9 ± 0.7	0.83 ± 0.05	7.18 ± 0.2	19.8 ± 0.75
*A. testudineus* (*N* = 15)	123.8 ± 31.8	17.4 ± 0.6	5.5 ± 0.3	72.3 ± 2.0	1.20 ± 0.10	9.28 ± 0.1	16.08 ± 0.61
*P. larnaudii* (*N* = 3)	708.6 ± 6.4	42.0 ± 1.0	10.0 ± 0.0	63.3 ± 0.3	0.51 ± 0.04	17.82 ± 0.04	17.2 ± 0.66
*O. niloticus* (*N* = 5)	291.0 ± 47.5	24.8 ± 1.3	8.2 ± 0.6	77.4 ± 0.5	2.49 ± 0.31	2.77 ± 1.3	17.5 ± 0.67
*C. micropeltes* (*N* = 2)	1348.5 ± 61.5	49.1 ± 0.2	11.0 ± 1.4	79.9 ± 0.7	0.76 ± 0.09	2.17 ± 0.06	16.39 ± 0.62
*C. batrachus* (*N* = 4)	550.0 ± 83.0	37.5 ± 5.7	7.7 ± 1.5	73.50	0.91 ± 0.04	9.12 ± 0.1	16.22 ± 0.62

*Note: Weight, length, and width were individually measured in each fish species.* *N* *= the number of fish to have 3* kg *of each fish species. Moisture and ash contents were measured in three samples per species from the pooled samples (* *n* = 3 *), whereas lipid and protein contents were measured in three samples per species from the pooled lyophilized samples (* *n* = 3 *).*

Before lyophilization, ground fish fillets were frozen at −20°C. After that, frozen samples were lyophilized using a freeze dryer machine (Christ, Alpha 1‐4 LD plus) at a temperature of −50°C and a pressure of 0.04 mbar for 44 ± 2 h [17]. After drying, the lyophilized samples were weighed, vacuumed, and stored at −20°C for further analysis.

### 2.2. Proximate Composition Analysis

Moisture and ash contents were measured in three samples per species from the pooled samples, whereas lipid and protein contents were measured in three samples per species from the pooled lyophilized samples.

Moisture content was analyzed following the AOAC method. Five grams of fish was put in aluminum caps and dried in an oven at 105°C ± 2°C until constant mass was achieved [19].

Ash content was analyzed by burning 5 g of a sample at 550°C for 3 h in a muffle furnace according to the AOAC method [19].

Proteins were analyzed by the Kjeldahl method [19]. Total protein content was calculated using the conversion factor of 6.25 [20].

The total lipid fraction was determined using a chloroform–methanol extraction procedure adapted from Folch et al. [21]. Briefly, 1 g of freeze‐dried material was homogenized with 1 mL of distilled water, vortex‐mixed, and left to equilibrate for 10 min. A solvent mixture of chloroform and methanol (2:1, v/v; 40 mL) was then added, and the suspension was placed on a rotary shaker and stirred continuously overnight. The resulting extract was passed through filter paper (MN615 ¼, Macherey‐Nagel) containing anhydrous sodium sulfate (> 99%) into 50‐mL Falcon tubes. Phase separation was induced by adding 8 mL of 0.88% KCl solution, followed by centrifugation at 3900 rpm for 10 min. After centrifugation, the upper aqueous–methanolic phase was carefully removed by aspiration. An aliquot of 10 mL from the organic phase was transferred to a clean glass tube and dried in an oven at 60°C until complete solvent evaporation. The residue was weighed, and lipid content was calculated gravimetrically. Results were expressed as grams of total fat per 100 g of fresh sample.

### 2.3. Mineral Content Analysis

Mineral contents were determined by the method described in Amoussou et al. [22]. Around 50 mg of the lyophilized sample was weighed into a Teflon digestion vessel within a closed microwave digestion system (EthosD, Milestone Inc.), employing hydrogen peroxide and nitric acid as reagents. The minerals were analyzed by inductively coupled plasma mass spectrometry with dynamic reaction cell technology (ICP‐MS ELAN DRC II, Perkin‐Elmer). Analytical quality control (QC) was ensured using certified reference materials (CRMs), such as DORM‐4 (fish protein; National Research Council, Canada) and GBW 07603 (bush branches and leaves; Chinese Institute of Geophysical and Geochemical Exploration). These CRMs were injected in each batch and subjected to the same digestion and instrumental analysis as the samples. The results obtained from the CRMs were in line with the certified values for all mineral contents. Additionally, the QC measures included procedural blanks and duplicates to monitor contamination and analytical precision (Table S1). Limits of detection (LOD) and quantification (LOQ) for each mineral were calculated from their specific blank distribution, both expressed as milligrams of element per kilogram of wet weight (mg/kg ww). Raw data were initially expressed as dry weight and then converted to wet weight by accounting for the samples′ moisture content. For quantification, five different concentrations of each standard element were used to build calibration curves for the quantification of each element composition in the samples.

### 2.4. Fatty Acid Analysis

The fatty acid profile was determined by analyzing the fatty acid methyl esters (FAMEs) by gas chromatography coupled to mass spectrometry (GC‐MS) [23]. The method consists of the methylation of the fat extracted from samples in the presence of the internal standard nonadecanoic acid (C19:0). FAMEs were separated on Focus GC gas chromatograph (Thermo Fisher Scientific) using a CP‐Sil88 column for FAME (100 × 0.25 mm, 0.2 *μ*m, Varian; Agilent Technologies, Santa Clara, California), analyzed with an ion trap PolarisQ mass spectrometer (Thermo Fisher Scientific). First, GC conditions were inlet temperature set at 250°C, with splitless injection, and helium used as the carrier gas at 1.5 mL/min, temperature program initiated at 55°C/min to 180°C, then 10°C/min to 200°C for 15 min, then an increase of 10°C/min to 225°C for 14 min; total run time was 59.50 min. The injection volume was 1 *μ*L. The MS conditions were transfer line set at 250°C and ion source at 220°C. The FAMEs were detected using selected ion monitoring (SIM) mode. The peaks identification was compared with their mass spectrum and retention times with those of the corresponding standards (C10:0 to C24:0). In each chromatographic run, two ions were monitored for each fatty acid analyzed, which facilitated detection and quantitative analysis: m/z 74 and 143 for SFA, 79 and 91 for monounsaturated fatty acids (MUFAs) and PUFA. The sum of SFA, MUFA, and PUFA was individually expressed as the percentage of the total fatty acids. For quantification, an eight‐point calibration curve was established for each of the 23 FAMEs using standard solutions containing known concentrations of the analytes and the internal standard (C19:0). The response factor was calculated as the ratio of the peak area of each FAME to that of the internal standard. Next, these response ratios were plotted against the standard concentrations, and linear regression analysis was applied without any fit weighting. The calibration curves showed good linearity across the tested concentration range. The calibration curve equations, relative retention time (RRT), ion ratio, and linearity (*R* ^2^) values are shown in Table S2. Fatty acids were considered positively identified in samples if the ratio between the chromatographic retention time of the analyte and that of the internal standard, that is, the relative retention time (RRT) of the analyte, corresponded to that of the average retention time of the calibration solutions within a ±0.5% tolerance and the peak area ratio of the two ions of each fatty acid corresponded to that of the averaged ion ratio of the calibration solutions within a tolerance of 20%, as set by the Commission Decision 2002/657/EC [24].

A QC sample, CRM BCR‐163 (beef‐pork fat blend, Sigma‐Aldrich), was used to assess the method′s performance (Table S3) and was analyzed with each series of samples.

### 2.5. NQI

The results from fatty acid profile determination were used to compute the lipid quality indices, using three indicators such as the thrombogenicity index (TI), atherogenicity index (AI), and the hypocholesterolemic/hypercholesterolemic (h/H) ratio according to the methods described by Chakma et al. [7] and Ulbricht and Southgate [25]. The NQI were calculated as follows:

1. *AI = (4 × C14:0 + C16:0 + C18:0)/(ΣMUFA + Σn-3 PUFA + Σn-6 PUFA)*
2. *TI = (C14:0 + C16:0 + C18:0)/((0.5 × ΣMUFA) + (0.5 × Σn-6PUFA) + (3 × Σn-3 PUFA) + (Σn-3 PUFA/Σn-6 PUFA))*
3. *h/H = (C18:1n-9 + C18:2n-6 + C20:4n-6 + C18:3n-3 + C20:5n3 + C22:5n3 + C22:6n3)/(C14:0 + C16:0)*

### 2.6. Nutritional Contribution of Essential Minerals and Semi‐Essential Fatty Acids

The nutritional intake of farmed freshwater fish consumption was estimated by considering the content of 10 dietary essential elements (Ca, Cu, Fe, K, Mn, Mo, Na, P, Se, and Zn) and the sum of semi‐essential fatty acids EPA (20:5 n‐3) and DHA (22:6 n‐3).

According to FAO, the average consumption of freshwater fish in Cambodia is 33 kg per year per person [3, 4, 26], which corresponds to an average of 90 g per day. The nutritional contribution was calculated based on the formula [22, 27] below:

4. Nutritional contribution (NC %)=[(C * M) * 100]/DRI

where *C* is the value of the essential elements (mg/kg); *M* is the meal portion consumed (g); and DRI is the dietary reference intake (mg) value established by the national academies, engineering, and medicine [28] and the European Food Safety Authority [29]. Regarding EPA and DHA, consumption for primary cardiovascular protection should be between 250 and 500 mg for adults [29]. In this study, the upper level of 500 mg/day was utilized to calculate the nutritional contribution of EPA + DHA.

### 2.7. Data Analysis

All the results were presented as mean ± standard deviation. The moisture content was used to convert from dried to fresh weight. To ensure the accuracy and traceability of the analysis method, CRMs were included in each analytical batch to verify the method. R software was utilized to analyze the results of this study. Principal component analysis (PCA) was employed to assess multivariate patterns among fish species, fatty acid profiles, and NQI variables. Before the PCA biplot, all variables were scaled to unit variance, and a heat map of the mineral concentrations for the farmed fish species was created. Mineral concentrations below their analytical LODs and LOQs were considered half of the LODs and LOQs during statistical data analysis [22]. Data on minerals were presented in natural log‐transformed form to meet the assumptions of standard parametric statistical tests, reduce the influence of outliers on the data distribution, and bring elemental concentrations into a common range. However, there is no statistical comparison among fish species in this study, as it aims to present compositional data descriptively to serve as baseline reference values.

## 3. Results

### 3.1. Morphology and Proximate Composition

The results of the morphology and proximate composition of the 10 farmed freshwater fish are demonstrated in Table 1. The average weight and length of the smallest fish species were 123.86 ± 31.89 g and 17.43 ± 0.67 cm, respectively. The average weight and length of the biggest fish species were 1348.50 ± 61.51 g and 49.15 ± 0.21 cm, respectively.

In this study, moisture, ash, lipid, and protein contents were analyzed in 10 different farmed freshwater fish species. Moisture content ranged from 63.37 ± 0.37 g/100 g (*P. larnaudii*) to 79.98 ± 0.72 g/100 g (*C. micropeltes*). Similarly, *P. larnaudii* presented the lowest ash content (0.51 ± 0.04 g/100 g) among the 10 farmed fish species. Meanwhile, *O. niloticus* showed the highest ash content (2.49 ± 0.31 g/100 g).

Lipid content in 10 farmed freshwater fish varied from 1.60 ± 0.1 to 17.82 ± 0.04 g/100 g wet weight. *P. larnaudii* presented the highest lipid content, whereas *L. rohita* had the lowest among the 10 fish species. In contrast to lipid results, *L. rohita* presented the highest protein content (21.91 g/100 g wet weight) compared to the other fish species. Overall, protein content ranged from 16.22 to 21.91 g/100 g wet weight for all farmed fish species.

### 3.2. Mineral Contents

**Figure 3:**
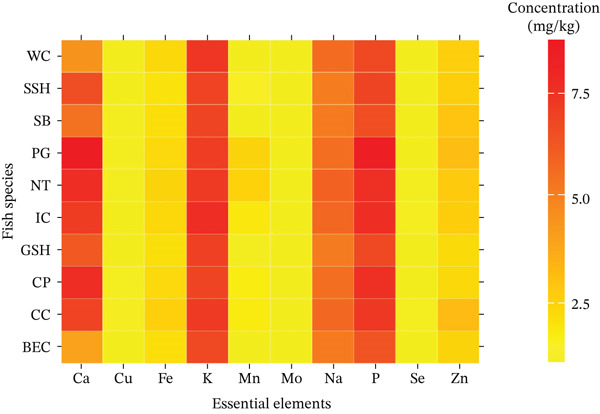
Heat map represents the essential element content such as potassium (K), phosphorus (P), sodium (Na), calcium (Ca), copper (Cu), iron (Fe), magnesium (Mn), molybdenum (Mo), selenium (Se), and zinc (Zn) in Cambodian farmed freshwater fish species, for *Clarias batrachus* (WC), *Channa striata* (SSH), *Barbonymus gonionotus* (SB), *Pangasius hypophthalmus* (PG), *Oreochromis niloticus* (NT), *Labeo rohita* (IC), *Channa micropeltes* (GSH), *Anabas testudineus* (CP), *Cyprinus carpio* (CC), and *Pangasius larnaudii* (BEC). Results were natural log‐transformed to create the figure.

The results of essential elements are presented in Figure 3 and Table S4. The heat map displays the essential element content, expressed as milligrams per kilogram (mg/kg) of wet weight, across 10 farmed freshwater fish species. Each cell represents the concentration of a specific element in a particular species, with color intensity ranging from yellow (lower level) to red (higher level), visually highlighting variation among species. In addition, essential elements such as potassium (K), phosphorus (P), sodium (Na), and calcium (Ca) were found as the major elements that were of high contents and have dark red color in the heat map, in contrast to minor elements such as copper (Cu), iron (Fe), magnesium (Mn), molybdenum (Mo), selenium (Se), and zinc (Zn) that were of low content and have yellow color in the heat map. Notably, *P. hypophthalmus* (PG) and *A. testudineus* (CP) exhibited the highest levels of Ca (8612 and 3735 mg/kg, respectively), whereas *L. rohita* (IC) showed the highest levels of K (3723 mg/kg). Similarly, minerals Na and P were more abundant in *O. niloticus* (NT) and *P. hypophthalmus* (PG), indicating that mineral accumulation is strongly influenced by fish species.

### 3.3. Fatty Acid Profile

Analysis of 29 fatty acids in the 10 different farmed freshwater fish species available in Cambodia revealed a consistent trend across most species (Table S5). SFAs dominated the profiles, ranging from 40.4 to 56.3 g/100 g total fatty acids, followed by PUFAs with levels between 26.4 and 51.5 g/100 g total fatty acids, whereas MUFAs were the least abundant (3.5–29.9 g/100 g total fatty acids).

Within SFAs, palmitic acid (C16:0) was the most prevalent, with *B. gonionotus* boasting the highest content (41.2 g/100 g total fatty acids) and *C. carpio* having the lowest (27 g/100 g total fatty acids). *P. larnaudii* exhibited the highest MUFA content (29.9 g/100 g total fatty acids), with oleic acid (C18:1 n‐9) as the dominant MUFA (24.3 g/100 g total fatty acids).

For n‐3 fatty acid, alpha‐linolenic acid (ALA, C18: n‐3), EPA (C20:5 n‐3), and DHA (C22:6 n‐3) exhibited the highest values for all the farmed freshwater fish species with a higher DHA level (0.9–14.9 g/100 g total fatty acids), followed by EPA (3.1–8.5 g/100 g total fatty acids), and ALA (1.3–6.1 g/100 g total fatty acids). Interestingly, *C. carpio* presented the highest DHA level (14.9 g/100 g total fatty acids), followed by *L. rohita* (13.6 g/100 g total fatty acids). In addition, the content of n‐6 PUFAs across species varied from 12.9 to 35.2 g/100 g total fatty acids, with linoleic acid (LA) (C18:2 n‐6) being the primary contributor, ranging from 6.2 to 29.8 g/100 g total fatty acids, which is the highest value found in *A. testudineus* (29.8 g/100 g total fatty acids).

### 3.4. NQI

The ratio of PUFA to SFA (PUFA/SFA), omega‐6/omega‐3 (n‐6/n‐3) ratio, AI, TI, and h/H were investigated to evaluate the lipid quality in farmed freshwater fish in this study. PUFA/SFA ratio varied from 0.5 to 1.34, and the ratio of n‐6/n‐3 ranged from 0.6 to 11.1. Furthermore, the AI and TI index values were calculated at values ranging from 0.5 to 1.13 and 0.39 to 1.24, respectively. The value of the h/H index in farmed fish species ranged from 0.63 to 1.76.

**Figure 4:**
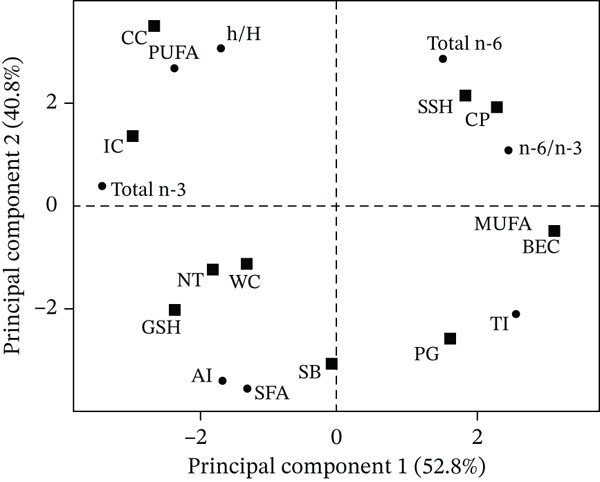
Principal component analysis (PCA) of total fatty acid profile and nutritional indices such as SFA: saturated fatty acid; MUFA: monounsaturated fatty acid; PUFA: polyunsaturated fatty acid; AI: atherogenicity index; TI: thrombogenicity index; and (h/H): hypocholesterolemic/hypercholesterolemic ratio in Cambodian farmed freshwater fish species, for *Clarias batrachus* (WC), *Channa striata* (SSH), *Barbonymus gonionotus* (SB), *Pangasius hypophthalmus* (PG), *Oreochromis niloticus* (NT), *Labeo rohita* (IC), *Channa micropeltes* (GSH), *Anabas testudineus* (CP), *Cyprinus carpio* (CC), and *Pangasius larnaudii* (BEC).

In addition, the PCA was performed to observe the relation between fish species and NQI and the total fatty acids (Figure 4). The first two principal components (PC1 and PC2) explained a 93.6% of the total variance, with PC1 contributing to 52.8%, and separated all the 10 farmed freshwater fish species depending on h/H, total n‐3, AI, SFA, total n‐6, n‐6/n‐3, TI, and MUFA. The second principal component (PC2) accounts for 40.8% of the variance. Meanwhile, PC1 and PC2 separated fish species into four clusters. This finding revealed that *C. carpio* (CC) and *L. rohita* (IC) showed higher levels of PUFA, total n‐3, and h/H ratio, leading to more favorable lipid profiles in terms of cardiovascular health. In contrast, other fish species such as *C. micropeltes* (GSH), *O. niloticus* (NT), and *B. gonionotus* (SB) were characterized by higher SFA percentages.

### 3.5. Nutritional Contribution of Essential Dietary Elements and Semi‐Essential Fatty Acids

In this study, the contribution of 10 essential dietary elements, such as Ca, Cu, Fe, Mn, Mo, P, K, Se, Na, and Zn, and the sum of EPA and DHA to the recommended nutritional daily intake were calculated (Table S6). Essential element concentrations could vary, depending on the consumer′s sex as well as the mineral concentrations in specific fish species. Among the nutritional contributions, the most contributing essential elements are calcium (0.39%–77.51%), followed by phosphorus (13.11%–75.73%), and selenium (14.37%–73.64%). In addition, based on the EPA + DHA measured in this study, for instance, walking catfish (*C. batrachus*), a 90‐g daily serving could supply an estimated 169.55% of the recommended daily intake (EPA + DHA).

## 4. Discussion

This study showed that species‐specific factors influenced the proximate composition, mineral levels, and fatty acid profiles of 10 commonly farmed freshwater fish in Cambodia. Among the proximate components, protein content ranged from 16.22 to 21.91 g/100 g, with *L. rohita* (IC) showing the highest levels, whereas fat content showed greater variability, with *P. larnaudii* accumulating the most lipids. Moisture and ash contents also differed among species, reflecting variation in body composition and growth characteristics. Overall, the nutritional profile of fish varied from species to species, regardless they are taxonomically related and cultured in similar ecosystems [8]. Moreover, fish feed is one of the main parameters that influence the nutritional composition of fish because different types of diets determine fish growth rate and flesh composition, especially lipid content [30] and fatty acid profiles [31]. In addition, exogenous factors such as environmental conditions and feed characteristics (type and size), together with feeding management practices (feeding frequency and feeding rate), influence feeding behavior and efficiency. These factors, along with the fish′s nutrient absorption capacity, ultimately affect growth performance and lipid composition [32, 33]. According to Tuttle et al. [34], feeding strategies significantly influence growth parameters and lipid composition in the largemouth bass (*Micropterus nigricans*) under high water temperature conditions.

In this study, the morphometric traits of farmed fish species were collected from different farms and provinces in Cambodia. These variations are likely influenced by factors including farm location, aquatic environment, water quality, temperature, and feeding behaviors, as reported in previous studies [35–37].

The proximate compositions of fish were influenced by a variety of factors, including species, age, habitat conditions (e.g., water temperature and salinity), feed type and availability, feeding strategies, and season [38]. In the current study, the moisture content in *P. hypophthalmus* was corroborated with the moisture reported in farmed *P. hypophthalmus* [7]. However, the results of moisture in *C. striata* and *C. micropeltes* found in this study were lower than the moisture reported in these species caught in natural habitats [17]. This difference may be explained by a negative correlation between fat and moisture content in fish. As lipid accumulates in muscle tissue, it leads to a decrease in moisture content in fish. Consequently, farmed fish with higher fat content tend to exhibit correspondingly lower moisture content. This is consistent with a previous study who reported that a reverse correlation between lipid and moisture content, where *Catla catla* had higher moisture 76.05% with lower fat 2.57%, whereas low moisture was detected in *L. rohita* 72.10% with higher fat 3.11% [11].

In general, fish were categorized based on their fat content into distinct groups such as lean fish (< 2% fat), low fat (2%–4% fat), medium fat (4%–8% fat), and high fat (> 8% fat) [39]. In the present study, two fish species, such as *L. rohita* and *A. testudineus* belong to the lean fish group. On the other hand, *B. gonionotus*, *C. batrachus*, and *P. larnaudii* are considered high‐fat fish (fat > 8%). The investigated farmed *C. striata* in this research were found to have higher fat content compared to the previous study (1.7 ± 0.3 g/100 g) for wild *C. striata* [17], and wild *C. micropeltes* was 0.25 ± 0.12 g/100 g [40]. This difference may be attributed to controlled conditions in fish farming, such as consistent water temperature and quality, and regular feeding. These controlled environments could optimize energy consumption and promote growth, potentially leading to higher fat accumulation compared to wild fish exposed to environmental variations and stresses and limited access to feed [30, 38, 41]. In addition, commercial feeds are generally high in fat content and dietary carbohydrate [38], which could contribute to a modified nutritional profile compared to wild‐caught fish.

The protein content in the 10 Cambodian farmed freshwater fish ranged from 16% to 21% on a wet basis. This variation in protein composition among farmed fish may be attributed to differences in feeding strategies [42]. For instance, Chakma et al. [7] reported that farmed *P. hypophthalmus* were fed artificial feed containing 30% protein. These fish had a higher protein content than wild *P. hypophthalmus* that were not fed nutrient‐rich artificial feed in a controlled environment. According to Wang et al. [32], feeding strategy was significantly influenced by protein content in *Schizothorax wangchiachii* by feeding one, two, three, and four times per day with a commercial diet that contained 33% crude protein and 6% lipids. Stansby [43] established that fish with a minimum protein level of 15% is classified as high protein fish. Furthermore, European Regulation No. 1924/2006 defines the criteria for food products to be labeled as “high protein” if at least 20% of the energy value of the food is provided by protein [44]. Hence, the Cambodian farmed freshwater fish in this study were considered to have high protein content. The high protein content of farmed freshwater fish species may play an important role in improving food and nutrition security in Cambodia. Fish with higher protein content provide more essential amino acids and could help meet daily protein requirements, particularly in low‐income households where fish is the primary and most affordable animal‐source food [2, 5]. Previous research has shown that giant snakeheads with a higher protein content (16.8%) contain greater amounts of essential amino acids, including lysine, methionine, leucine, isoleucine, and valine, than individuals of the same species with lower protein content (13.7%) [45]. Therefore, the high protein and essential amino acid profiles observed in the studied fish species are nutritionally significant, as essential amino acids cannot be synthesized by the human body and must be obtained through diet. These characteristics highlight their potential as accessible and locally available protein sources, contributing to improved nutritional status and supporting public health.

The ash content in the analyzed fish species ranged from 0.51*%* ± 0.04*%* to 2.49*%* ± 0.31*%*. This variation reflects differences in total mineral composition among species. Indeed, a previous study observed an increase in the ash content within the edible part (fish fillets) of certain fish species when bones were present [46]. In the present study, *O. niloticus* exhibited the highest ash content (2.49*%* ± 0.31*%*), which could be related to the muscle‐to‐bone ratio, and residue bone fragments may partially contribute to ash content. However, calcium concentration was the highest in pangasius, suggesting that ash content does not solely reflect the calcium level. If the filleting and deboning were not entirely thorough, residual bones might have contributed to the higher ash content.

The predominant essential elements detected in the studied farmed fish species were calcium (Ca), potassium (K), phosphorus (P), and sodium (Na), which were present at substantially higher levels than the trace elements iron (Fe), zinc (Zn), molybdenum (Mo), manganese (Mn), selenium (Se), and copper (Cu). This is consistent with previous findings reported by Islam et al. [15]. Moreover, from a nutritional perspective, a higher potassium (K) and lower sodium (N) content is considered beneficial, as diets with a favorable Na/K ratio are associated with reduced risk of hypertension and cardiovascular diseases. As claimed by Bu et al. [47], a higher dietary Na/K ratio has been associated with unfavorable serum lipid profiles and increased cardiovascular risk, whereas a lower Na/K ratio is considered beneficial for cardiovascular health. The observed interspecies differences in both macro and microelements may be attributed to biological and environmental factors, including age, body size, feeding practices, habitat conditions, and aquaculture systems, as previously described by Njinkoue et al. [48].

In addition to proximate composition and essential minerals, the quality and profile of fatty acids further define their contribution to human health. The results of fatty acid compositions showed that there were considerable differences among the farmed fish species in this study. These results may be explained by the fact that the fatty acid composition was affected by several variables, including the species, fish size and age, seasonal conditions, geographic location, and type of feed [11, 31].

This current study showed that palmitic acid (C16:0), oleic acid (C18:1 n‐9), and LA (C18:2n‐6) were the main compounds of SFAs, MUFAs, and PUFAs, respectively. This result is similar to what was observed in previous studies [11, 17, 49–51].

In addition, the fatty acid composition of fish fillets, particularly the levels of palmitic acid (C16:0), oleic acid (C18:1 n‐9), LA (C18:2 n‐6), ALA (C18:3 n‐3), EPA (C20:5 n‐3), and DHA (C22:6 n‐3), is strongly influenced by the composition of the feed provided during aquaculture [12]. However, the fatty acid composition in farmed fish is influenced not only by the feed ingredients but also by species‐specific metabolic traits, including their capacities for fatty acid chain elongation and desaturation [52, 53]. This finding revealed that the variation of fatty acid compositions of each Cambodian‐farmed freshwater fish species is probably attributed to the feed used in each farm. This result is similar to what was observed in previous studies [11, 17, 49–51]. Furthermore, Cambodian farmers reportedly use diverse feed types, including Moina, pelleted feed, trash feed, rice bran, and plant materials such as pumpkin. Such variability in feed composition likely contributes to the observed differences in fatty acid profiles among species. Plant‐derived ingredients such as rice bran are rich in LA (C18:2 n‐6) and ALA (C18:3 n‐3), which serve as precursors for long‐chain polyunsaturated fatty acids (LC‐PUFAs). Freshwater fish species possess Δ5, Δ6 desaturase and elongase enzymes that enable the bioconversion of C18 precursors into C20 and C22 fatty acids, such as EPA and DHA, although conversion efficiency is species specific [12, 52]. In this study, EPA and DHA were the dominant n‐3 fatty acids. *L. rohita* exhibited the highest EPA content (8.5 g/100 g of total fatty acids), nearly double what has been observed in *C. carpio* and *C. batrachus* (4.4 g/100 g of total fatty acids), and substantially higher than in *B. gonionotus* (3.1 g/100 g of total fatty acids). In contrast, DHA levels were the highest in *C. carpio* (14.9 g/100 g of total fatty acids), closely followed by *L. rohita* (13.6 g/100 of total fatty acids), whereas lower concentration were detected in *B. gonionotus* (6 g/100 g of total fatty acids) and *C. batrachus* (5.8 g/100 g of total fatty acids). These interspecific differences in EPA and DHA levels suggest variability in endogenous elongation and desaturation activity, as well as potential differences in feed composition and lipid metabolism among species, particularly the balance between plant‐ and animal‐derived lipid sources. Therefore, both species biology and farm‐level feeding strategies likely contribute to the distinct n‐3 LC‐PUFA profiles observed. This result is consistent with previous studies that reported that a higher amount of LA in farmed fish is related to the feed ingredients and chain elongation and desaturation capacity [11], and a high concentration of DHA in muscle of farmed yellow perch could have been influenced by the content of fish meal in the commercial diet [31].

In general, most of the investigated fish species had a value of n‐6/n‐3 of less than 4, and the ratio of PUFA/SFA was at least 0.45, which is recommended for the prevention of cardiovascular disease [13]. The PUFA/SFA and n‐6/n‐3 ratios, which are used to evaluate the nutritional value of lipids, only produce basic dietary recommendations since they both fail to consider MUFA [17]. The values of the indices, such as AI, TI, and h/H, were calculated to better characterize the lipid quality of the 10 farmed freshwater fish species. AI and TI indices, originally introduced by Ulbricht and Southgate [25], are designed to assess how dietary fats may affect the risk of coronary disease. High AI and TI levels may increase platelet aggregation, resulting in thrombus and atheroma development in the cardiovascular system. Thus, lower AI and TI levels are beneficial to avoid cardiovascular diseases [13]. The fatty acids involved in cholesterol metabolism are taken into consideration by h/H, and higher h/H levels are thought to be better for human health [13, 41]. The results of this study showed similar values of AI, TI, and h/H, except for some fish species such as *B. gonionotus* (SB), *P. larnaudii* (BEC), and *P. hypophthalmus* (PG), which had higher AI and TI values, and lower h/H compared to the studied species. However, their values remain within the ranges commonly reported for freshwater fish [7, 12]. Therefore, some species showed less favorable lipid quality indices; their overall nutritional quality remains beneficial, particularly when consumed as part of a balanced diet rich in unsaturated fatty acids, high‐quality protein, and essential minerals. This difference may be due to the impact of fish diets. Therefore, foods with lower AI, TI, and high h/H scores are considered to have healthier lipid profiles and a greater potential to contribute to the prevention of cardiovascular disorders.

This study assessed the nutritional value of farmed fish species using the guidelines provided by EFSA, with a daily maximum dietary intake of 500 mg of EPA and DHA to prevent primary cardiovascular diseases. It was observed in the current investigation that four fish species met these requirements, namely *C. batrachus* (WC), *B. gonionotus* (SB), *C. carpio* (CC), and *A. testudineus* (CP) [29]. Indeed, the results showed that consuming 90 g of the studied farmed freshwater fish, especially *C. batrachus*, *B. gonionotus*, *C. carpio*, and *A. testudineus* per capita per day, could provide up to 169.55%, 140.08%, 109.14%, and 109.14%, respectively, of the daily required intake for semi‐essential fatty acids (EPA + DHA). Consequently, these findings exhibit the nutritional potentials of Cambodian‐farmed fish species, especially in terms of their fatty acid profiles, advantaging Cambodian human health and promoting the valorization of farmed freshwater fish as a sustainable alternative to wild‐caught fish, whose populations are experiencing documented declines.

For further research, it is recommended to investigate the impact of environmental parameters (water quality, farming conditions, and seasonal variation) and feed compositions used in aquaculture systems to provide a more comprehensive understanding of factors shaping fish nutritional value. In addition, although this study provides comprehensive baseline compositional data for Cambodian farmed freshwater fish species, it should take into account applying a statistical comparison among fish species to identify which species are superior in terms of nutritional value.

## 5. Conclusion

This study highlights the potential health benefits of consuming Cambodian farmed freshwater fish species. All investigated fish offered a valuable source of protein, essential dietary elements, and a range of fatty acids, including ALA, LA, EPA, and DHA. Moreover, consuming individually walking catfish (*C. batrachus*) or in combination with other studied farmed fish species could provide a substantial amount of EPA and DHA, sufficient to meet or exceed the recommended daily intake for these essential fatty acids. EPA and DHA were detected in all studied species, although their relative proportions differed considerably, particularly *L. rohita*, *C. carpio*, *C. batrachus*, and *B. gonionotus* exhibited favorable n‐3 LC‐PUFAs. In addition, the fish provided essential macro and microelements, including Ca, K, P, Na, Fe, Zn, Mo, Mn, Se, and Cu, contributing to their overall nutritional value.

To sum up, promoting the consumption of these nutrient‐rich fish can contribute significantly to improving the overall nutritional profile of the population. Dietary diversification through increased farmed fish consumption could help address potential nutrient deficiencies and contribute to improved public health for the Cambodian people.

## Funding

This research was funded by Académie de recherche et d′enseignement supérieur (ARES), Belgium.

## Conflicts of Interest

The authors declare no conflicts of interest.

## Supporting information


**Supporting Information.** Additional supporting information can be found online in the Supporting Information section. Supplementary material related to this article can be found in the online version.

## Data Availability

The data that support the findings of this study are available from the corresponding author upon reasonable request.

## References

[1] Food and Agriculture Organization , In Brief to the State of World Fisheries and Aquaculture 2024, 2024, Blue Transformation in Action, 10.4060/cd0690en.

[2] Joffre O. M. , Freed S. , Bernhardt J. , Teoh S. J. , Sambath S. , and Belton B. , Assessing the Potential for Sustainable Aquaculture Development in Cambodia, Frontiers in Sustainable Food Systems. (2021) 5, 704320, 10.3389/fsufs.2021.704320.

[3] Food and Agriculture Organization , Calculated From Food Balance Sheets, 2019, FAOSTAT, accessed June 1, 2024, http://faostat.fao.org/site/368/DesktopDefault. aspx?PageID5368#ancor.

[4] Food and Agriculture Organization , National Aquaculture Sector Overview Cambodia, 2019, Fisheries and Aquaculture, accessed June 1, 2024, https://www.fao.org/fishery/en/countrysector/naso_cambodia.

[5] WorldFish , WorldFish in Cambodia, 2024, https://worldfishcenter.org/publication/worldfish-cambodia-0.

[6] Ashraf S. A. , Adnan M. , Patel M. , Siddiqui A. J. , Sachidanandan M. , Snoussi M. , and Hadi S. , Fish-Based Bioactives as Potent Nutraceuticals: Exploring the Therapeutic Perspective of Sustainable Food From the Sea, Marine Drugs. (2020) 18, no. 5, 10.3390/md18050265, 32443645. PMC7281228 32443645

[7] Chakma S. , Rahman M. A. , Siddik M. A. B. , Hoque M. S. , Islam S. M. , and Vatsos I. N. , Nutritional Profiling of Wild (Pangasius pangasius) and Farmed (Pangasius hypophthalmus) Pangasius Catfish With Implications to Human Health, Fishes. (2022) 7, no. 6, 10.3390/fishes7060309.

[8] Mohanty B. P. , Mahanty A. , Ganguly S. , Mitra T. , Karunakaran D. , and Anandan R. , Nutritional Composition of Food Fishes and Their Importance in Providing Food and Nutritional Security, Food Chemistry. (2019) 293, 561–570, 10.1016/j.foodchem.2017.11.039, 2-s2.0-85034823576, 31151648. 31151648

[9] NurSyahirah S. and Rozzamri A. , Effects of Frying on Fish, Fish Products and Frying Oil – A Review, Food Research. (2022) 6, no. 5, 14–32, 10.26656/fr.2017.6(5).608.

[10] Oluwaniyi O. O. , Dosumu O. O. , and Awolola G. V. , Effect of Local Processing Methods (Boiling, Frying and Roasting) on the Amino Acid Composition of Four Marine Fishes Commonly Consumed in Nigeria, Food Chemistry. (2010) 123, no. 4, 1000–1006, 10.1016/j.foodchem.2010.05.051, 2-s2.0-77955013029.

[11] Memon N. N. , Talpur F. N. , Bhanger M. I. , and Balouch A. , Changes in Fatty Acid Composition in Muscle of Three Farmed Carp Fish Species (Labeo rohita, Cirrhinus mrigala, Catla catla) Raised Under the Same Conditions, Food Chemistry. (2011) 126, no. 2, 405–410, 10.1016/j.foodchem.2010.10.107, 2-s2.0-78650677819.

[12] Rodrigues B. L. , Monteiro M. L. G. , Da Cruz V. , Silva Canto A. C. , Costa M. P. D. , and Conte-Junior C. A. , Proximate Composition, Fatty Acids and Nutritional Indices of Promising Freshwater Fish Species From Serrasalmidae Family, CyTA - Journal of Food. (2020) 18, no. 1, 591–598, 10.1080/19476337.2020.1804463.

[13] Zhang W. , Chen J. , Yue Y. , Zhu Z. , Liao E. , and Xia W. , Modelling the Mass Transfer Kinetics of Battered and Breaded Fish Nuggets During Deep-Fat Frying at Different Frying Temperatures, Journal of Food Quality. (2020) 2020, e8874163, 10.1155/2020/8874163.

[14] Gladyshev M. I. , Sushchik N. N. , Tolomeev A. P. , and Dgebuadze Y. Y. , Meta-analysis of Factors Associated With Omega-3 Fatty Acid Contents of Wild Fish, Reviews in Fish Biology and Fisheries. (2018) 28, no. 2, 277–299, 10.1007/s11160-017-9511-0, 2-s2.0-85038121217.

[15] Islam S. , Bhowmik S. , Majumdar P. R. , Srzednicki G. , Rahman M. , and Hossain M. A. , Nutritional Profile of Wild, Pond-, Gher- and Cage-Cultured Tilapia In Bangladesh, Heliyon. (2021) 7, no. 5, e06968, 10.1016/j.heliyon.2021.e06968, 34027173. 34027173 PMC8121654

[16] Mekonnen M. F. , Desta D. T. , Alemayehu F. R. , Kelikay G. N. , and Daba A. K. , Evaluation of Fatty Acid-Related Nutritional Quality Indices in Fried and Raw Nile Tilapia, (Oreochromis niloticus), Fish Muscles, Food Science & Nutrition. (2020) 8, no. 9, 4814–4821, 10.1002/fsn3.1760, 32994943. 32994943 PMC7500769

[17] Sroy S. , Arnaud E. , Servent A. , In S. , and Avallone S. , Nutritional Benefits and Heavy Metal Contents of Freshwater Fish Species From Tonle Sap Lake With Sain and Lim Nutritional Score, Journal of Food Composition and Analysis. (2021) 96, 103731, 10.1016/j.jfca.2020.103731.

[18] Lay S. , Phat C. , Mith H. , Scippo M.-L. , Mom V. , and Douny C. , Assessment of Fatty Acid Profiles and Proximate Composition of Ten Cambodian Farmed Freshwater Fish, 2025, SSRN, 10.2139/ssrn.5097239. PMC13212264 42212186

[19] Association of Official Analytical Chemists , Official Methods of Analysis of AOAC International, 2012, 19th edition, Association of Official Analytical Chemists.

[20] Food and Agriculture Organization , Food energy: Methods of Analysis and Conversion Factors, FAO Food and Nutrition Paper No. 77, 2003, FAO, https://openknowledge.fao.org/items/ccc3eb69-47e3-408d-8efb-9d3f68f637d2.

[21] Folch J. , Lees M. , and Stanley G. H. S. , A Simple Method for the Isolation and Purification of Total Lipides From Animal Tissues, Journal of Biological Chemistry. (1957) 226, no. 1, 497–509, 10.1016/S0021-9258(18)64849-5, 13428781. 13428781

[22] Amoussou N. , Marengo M. , Durieux E. D. H. , Douny C. , Scippo M.-L. , and Gobert S. , Trace Elements and Fatty Acid Profile of Argyrosomus regius (Asso, 1801) From Mediterranean Aquaculture, Biological Trace Element Research. (2020) 196, no. 2, 618–628, 10.1007/s12011-019-01925-x, 31625052. 31625052

[23] Douny C. , El Khoury R. , Delmelle J. , Brose F. , Degand G. , Moula N. , Farnir F. , Clinquart A. , Maghuin-Rogister G. , and Scippo M.-L. , Effect of Storage and Cooking on the Fatty Acid Profile of Omega-3 Enriched Eggs and Pork Meat Marketed in Belgium, Food Science & Nutrition. (2015) 3, no. 2, 140–152, 10.1002/fsn3.197, 2-s2.0-85051722692, 25838892. 25838892 PMC4376408

[24] European Commission , Commission Decision No 2002/657 of 14 August 2002 Implementing Council Directive 96/23/Ec Concerning the Performance of Analytical Methods and the Interpretation of Results, Official Journal of the European Union, L. (2002) 221.

[25] Ulbricht T. L. V. and Southgate D. A. T. , Coronary Heart Disease: Seven Dietary Factors, Lancet. (1991) 338, no. 8773, 985–992, 10.1016/0140-6736(91)91846-M, 2-s2.0-0025950886. 1681350

[26] Douny C. , Mith H. , Igout A. , and Scippo M.-L. , Fatty Acid Intake, Biogenic Amines and Polycyclic Aromatic Hydrocarbons Exposure Through the Consumption of Nine Species of Smoked Freshwater Fish from Cambodia, Food Control. (2021) 130, 108219, 10.1016/j.foodcont.2021.108219.

[27] Costa S. , Afonso C. , Bandarra N. M. , Gueifão S. , Castanheira I. , Carvalho M. L. , Cardoso C. , and Nunes M. L. , The Emerging Farmed Fish Species Meagre (Argyrosomus regius): How Culinary Treatment Affects Nutrients and Contaminants Concentration and Associated Benefit-Risk Balance, Food and Chemical Toxicology. (2013) 60, 277–285, 10.1016/j.fct.2013.07.050, 2-s2.0-84882760161, 23900006. 23900006

[28] Food and Nutrition Board , Dietary Reference Intakes (Dris): Recommended Intakes for Individuals, 2019, Food and Nutrition Board, https://www.ncbi.nlm.nih.gov/books/NBK545442/table/appJ_tab3/?report=objectonly.

[29] European Food Safety Authority , Scientific Opinion on Dietary Reference Values for Fats, Including Saturated Fatty Acids, Polyunsaturated Fatty Acids, Monounsaturated Fatty Acids, Trans Fatty Acids, and Cholesterol, EFSA Journal. (2010) 8, no. 3, 1461, 10.2903/j.efsa.2010.1461.

[30] Fuentes A. , Fernández-Segovia I. , Serra J. A. , and Barat J. M. , Comparison of Wild and Cultured Sea Bass (Dicentrarchus labrax) Quality, Food Chemistry. (2010) 119, no. 4, 1514–1518, 10.1016/j.foodchem.2009.09.036, 2-s2.0-70449651714.

[31] González S. , Flick G. J. , O’Keefe S. F. , Duncan S. E. , McLean E. , and Craig S. R. , Composition of Farmed and Wild Yellow Perch (Perca flavescens), Journal of Food Composition and Analysis. (2006) 19, no. 6–7, 720–726, 10.1016/j.jfca.2006.01.007, 2-s2.0-33744794306.

[32] Wang C. , Xie S. , Zheng H. , Chen F. , and Fang Y. , Effects of Feeding Frequency on the Growth, Body Composition and Sod, Gpx And Hsp70 Gene Expression in Schizothorax wangchiachii, Aquaculture Reports. (2022) 22, 100942, 10.1016/j.aqrep.2021.100942.

[33] Xie F. , Ai Q. , Mai K. , Xu W. , and Ma H. , The Optimal Feeding Frequency of Large Yellow Croaker (Pseudosciaena crocea, Richardson) Larvae, Aquaculture. (2011) 311, no. 1–4, 162–167, 10.1016/j.aquaculture.2010.12.005, 2-s2.0-78651500968.

[34] Tuttle J. T. , Smith M. A. , Roy L. A. , Jones M. , Lochmann R. , and Kelly A. M. , Effects of Different Feeding Regimes on Growth Rates and Fatty Acid Composition of Largemouth Bass Micropterus nigricans at High Water Temperatures, Animals. (2022) 12, no. 20, 10.3390/ani12202797, 36290183. PMC9597755 36290183

[35] Kwikiriza G. , Yegon M. J. , Byamugisha N. , Beingana A. , Atukwatse F. , Barekye A. , Nattabi J. K. , and Meimberg H. , Morphometric Variations of Nile Tilapia (Oreochromis niloticus) (Linnaeus, 1758) Local Strains Collected from Different Fish Farms in South Western Highland Agro-Ecological Zone (SWHAEZ), Uganda: Screening Strains for Aquaculture, Fishes. (2023) 8, no. 4, 10.3390/fishes8040217.

[36] Mariotto S. , Moriely Ramos Prado R. , Nascimento E. , Rodrigues De Souza X. , and De Abreu Sousa D. , Proximate and Fatty Acid Compositions oft en Wild-Caught and Farmed Fish Species in Mato Grosso State, Brazil, Chemistry & Biodiversity. (2024) 21, no. 2, e202301568, 10.1002/cbdv.202301568, 38252918. 38252918

[37] Medeiros Melo D. , Ferreira Roseno T. , Barros W. M. , De Faria R. A. P. G. , De Souza Paglarini C. , Bitencourt Faria P. , Mariotto S. , and De Souza X. R. , Fatty Acid Profiles and Cholesterol Content of Five Species of Pacu-Pevas From the Pantanal Region of Mato Grosso, Brazil, Journal of Food Composition and Analysis. (2019) 83, 103283, 10.1016/j.jfca.2019.103283, 2-s2.0-85070871586.

[38] Wang Y. , Yu S. , Ma G. , Chen S. , Shi Y. , and Yang Y. , Comparative Study of Proximate Composition and Amino Acid in Farmed and Wild Pseudobagrus ussuriensis Muscles, International Journal of Food Science & Technology. (2014) 49, no. 4, 983–989, 10.1111/ijfs.12391, 2-s2.0-84900651386.

[39] Mohanty B. P. , Ganguly S. , Mahanty A. , Sankar T. V. , Anandan R. , Chakraborty K. , Paul B. N. , Sarma D. , Syama Dayal J. , Venkateshwarlu G. , and Mathew S. , DHA and EPA Content and Fatty Acid Profile of 39 Food Fishes From India, BioMed Research International. (2016) 2016, 4027437, 10.1155/2016/4027437, 2-s2.0-84985021902, 27579313. 27579313 PMC4989070

[40] Chor L. , Sroy S. , Peng C. , and Doeurn S. , Process Optimization and Quality Assessment of Nem, a Traditional Cambodian Lactic Acid Fermented Fish Product, Journal of Food Science and Nutrition Research. (2024) 7, no. 1, 10.26502/jfsnr.2642-110000152.

[41] Petenuci M. E. , Rocha I. D. N. A. , De Sousa S. C. , Schneider V. V. A. , Da Costa L. A. M. A. , and Visentainer J. V. , Seasonal Variations in Lipid Content, Fatty Acid Composition and Nutritional Profiles of Five Freshwater Fish From the Amazon Basin, Journal of the American Oil Chemists′ Society. (2016) 93, no. 10, 1373–1381, 10.1007/s11746-016-2884-8, 2-s2.0-84982310431.

[42] Khan M. S. , Ang K. J. , Ambak M. A. , and Saad C. R. , Optimum Dietary Protein Requirement of a Malaysian Freshwater Catfish, Mystus nemurus, Aquaculture. (1993) 112, no. 2–3, 227–235, 10.1016/0044-8486(93)90448-8, 2-s2.0-38249003773.

[43] Stansby M. E. , Chemical Characteristics of Fish Caught in the Northeast Pacific Ocean, 1976, International Atomic Energy Agency.

[44] European Commission , Regulation (EC) No 1924/2006 of the European Parliament and of the Council of 20 December 2006 on Nutrition and Health Claims Made on Foods, Official Journal of the European Union, L. (2006) 404, 9–25, https://eur-lex.europa.eu/legal-content/EN/TXT/?uri=CELEX%253A32006R1924.

[45] Pratama W. W. , Nursyam H. , Hariati A. M. , Islamy R. A. , and Hasan V. , Short Communication: Proximate Analysis, Amino Acid Profile and Albumin Concentration of Various Weights of Giant Snakehead (Channa Micropeltes) From Kapuas Hulu, West Kalimantan, Indonesia, Biodiversitas Journal of Biological Diversity. (2020) 21, no. 3, 10.13057/biodiv/d210346.

[46] Bogard J. R. , Thilsted S. H. , Marks G. C. , Wahab M. A. , Hossain M. A. R. , Jakobsen J. , and Stangoulis J. , Nutrient Composition of Important Fish Species in Bangladesh and Potential Contribution to Recommended Nutrient Intakes, Journal of Food Composition and Analysis. (2015) 42, 120–133, 10.1016/j.jfca.2015.03.002, 2-s2.0-84928316740.

[47] Bu S.-Y. , Kang M.-H. , Kim E.-J. , and Choi M.-K. , Dietary Intake Ratios of Calcium-to-Phosphorus and Sodium-to-Potassium Are Associated With Serum Lipid Levels in Healthy Korean Adults, Preventive Nutrition and Food Science. (2012) 17, no. 2, 93–100, 10.3746/pnf.2012.17.2.093, 2-s2.0-84864853247, 24471069. 24471069 PMC3866749

[48] Njinkoue J. M. , Gouado I. , Tchoumbougnang F. , Ngueguim J. H. Y. , Ndinteh D. T. , Fomogne-Fodjo C. Y. , and Schweigert F. J. , Proximate Composition, Mineral Content and Fatty Acid Profile of Two Marine Fishes From Cameroonian Coast: Pseudotolithus typus (Bleeker, 1863) and Pseudotolithus elongatus (Bowdich, 1825), NFS Journal. (2016) 4, 27–31, 10.1016/j.nfs.2016.07.002, 2-s2.0-84981748153.

[49] Akter M. , Faruque H. , Hasan M. , and Rahman M. S. , Proximate Composition, Amino Acids and Fatty Acids Profiles of Wild and Cultured Climbing Perch, Anabas testudineus (Bloch, 1795), Bangladesh Journal of Zoology. (2021) 48, no. 2, 365–378, 10.3329/bjz.v48i2.52376.

[50] Sokamte T. A. , Mbougueng P. D. , Mohammadou B. A. , Tatsadjieu N. L. , and Sachindra N. M. , Proximal Composition and Fatty Acid Profile of Fresh and Smoked Fillets of Pangasius hypophthalmus, Scientific African. (2020) 9, e00534, 10.1016/j.sciaf.2020.e00534.

[51] Zuraini A. , Somchit M. N. , Solihah M. H. , Goh Y. M. , Arifah A. K. , Zakaria M. S. , Somchit N. , Rajion M. A. , Zakaria Z. A. , and Mat Jais A. M. , Fatty Acid and Amino Acid Composition of Three Local Malaysian Channa spp. Fish, Food Chemistry. (2006) 97, no. 4, 674–678, 10.1016/j.foodchem.2005.04.031, 2-s2.0-31844433091.

[52] Mellery J. , Brel J. , Dort J. , Geay F. , Kestemont P. , Francis D. S. , Larondelle Y. , and Rollin X. , A n-3 PUFA Depletion Applied to Rainbow Trout Fry (Oncorhynchus mykiss) Does Not Modulate Its Subsequent Lipid Bioconversion Capacity, British Journal of Nutrition. (2017) 117, no. 2, 187–199, 10.1017/S0007114516004487, 2-s2.0-85010840084, 28112058. 28112058 PMC5314960

[53] Nguyen Q. V. , Malau-Aduli B. S. , Cavalieri J. , Nichols P. D. , and Malau-Aduli A. E. O. , Enhancing Omega-3 Long-Chain Polyunsaturated Fatty Acid Content of Dairy-Derived Foods for Human Consumption, Nutrients. (2019) 11, no. 4, 10.3390/nu11040743, 2-s2.0-85064197411, 30934976. PMC6520953 30934976

