# Supplementary material for: Assessment of Fatty Acid Profiles and Proximate Composition of 10 Cambodian Farmed Freshwater Fish
Source: Int J Food Sci. 2026 May 26;2026:5927997. doi: 10.1155/ijfo/5927997 (PMC13212264; doi:10.1155/ijfo/5927997)
Supplement: Supplementary file 1 — Supporting Information. Additional supporting information can be found online in the Supporting Information section. Supplementary material related to this article can be found in the online version. [file IJFO-2026-5927997-s001.pdf]

Supplementary material

Table S1. Calibration curve equation for minerals

**Elements**	**Calibration curve equation**	**Linearity (R^2)**
Ca	$Y= 1.484x10^{-3} X+4.441x10^{-2}$	0.9993
Cu	$Y= 3.546x10^{-2} X+1.675x10^{-4}$	1.0000
Fe	$Y= 2.338x10^{-2} X+7.092x10^{-3}$	1.0000
K	$Y= 5.078x10^{-2} X+6.118$	1.0000
Mn	$Y= 1.531x10^{-2} X+5.909x10^{-5}$	1.0000
Mo	$Y= 2.081x10^{-2} X+1.622x10^{-5}$	1.0000
Na	$Y=3.252x10^{-3} X+1.211x10^{-1}$	0.9999
P	$Y= 5.343x10^{-5} X+1.413x10^{-3}$	0.9999
Se	$Y= 5.843x10^{-4} X+4.971x10^{-6}$	1.0000
Zn	$Y= 5.252x10^{-3} X+3.374x10^{-4}$	1.0000

Table S2. Calibration curve equation for fatty acids

Fatty acids	Calibration curve equation	Linearity (R^2)	Relative retention time	Ion ratio	LOD	LOQ
DEME (C10:0)	Y=0.0421X-0.0006	0.9974	0.60	2.56	0.3	0.6
DOME (C12:0)	Y=0.0376X+0.0031	0.9963	0.70	2.60	0.1	0.3
TRIME (C13:0)	Y=0.0199X-0.0033	0.9972	0.74	2.44	0.4	0.8
MYRME (C14:0)	Y=0.0469X+0.0197	0.9981	0.79	2.77	0.1	0.3
PALME (C16:0)	Y=0.0522X+0.0459	0.9951	0.89	2.33	0.3	0.7
HPME (C17:0)	Y=0.0407X-0.0016	0.9967	0.91	2.36	0.1	0.3
STEME (C18:0)	Y=0.0571X-0.0008	0.9852	0.96	2.70	0.2	0.5
ARIME (C20:0)	Y=0.0341X-0.0041	0.9898	1.04	2.77	0.1	0.3
DCME (C22:0)	Y=0.0339X-0.0011	0.9991	1.12	1.84	0.1	0.3
TCME (C24:0)	Y=0.0239X+0.0003	0.9997	1.21	1.82	0.1	0.3
POME (C16:1)	Y=0.0227X+0.0129	0.9996	0.91	2.36	0.1	0.3
HDME (C17:1)	Y= 0.0196X+0.0015	0.9968	0.95	2.29	0.4	0.9
TEME (C18:1,9t)	Y=0,0312X+0.0668	0.9978	0.98	2.41	0.2	0.4
OLME (C18:1,9c)	Y=0.0458X+0.35	0.9987	0.99	2.25	0.5	1.2
CVME (C18:1,11c)	Y=0.0404X+0.0129	0.9895	0.99	2.45	0.2	0.4
ERME (C22:1)	Y=0.0166X-0.0014	0.9973	1.15	2.16	0.2	0.4
LAME (C18:2,9c,12c)	Y=0.0497X+0.0096	0.997	1.03	3.03	0.3	0.6
LDME (C18:2,9t,12t)	Y=0.0177X-0.0023	0.9943	1.01	2.82	0.2	0.4

RMME (C18:2,9c,11t)	Y=0.009X+0.0047	0.9767	1.08	3.00	0.2	0.4
ILME (C18:2,9t,11t)	Y=-0.0005X+0.0029	0.0852	1.08	2.96	0.5	1.2
GLAME (C18:3 (n-6))	Y=0.06X-0.0046	0.9943	1.05	2.49	0.1	0.3
ED6ME (C20:2)	Y=0.1215X-0.0108	0.9915	1.10	2.28	0.1	0.3
ARAME (C20:4)	Y=0.052X-0.0082	0.9947	1.16	1.93	0.2	0.5
ALAME (C18:3 (n-3))	Y=0.0571X-0.0069	0.9945	1.07	2.84	0.2	0.5
SAME (C18:4)	Y=0.0677X+0.0324	0.9961	1.10	3.05	0.1	0.3
ETME (C20:3)	Y=0.0587X-0.0051	0.9968	1.16	2.89	0.2	0.5
EPAME (C20:5)	Y=0.0126X-0.0006	0.9972	1.23	1.82	0.2	0.5
DPAME (C22:5)	Y=0.0316X-0.002	0.9971	1.36	1.89	0.2	0.5
DHAME (C22:6)	Y=0.0221X-0.0009	0.998	1.41	1.69	0.2	0.5

Table S3. Performance parameters for fatty acid analysis by GC-MS

CRM (BCR-163, beef pork fat blend)								
Fatty acids		Relative mass fraction in g FAME/100g total FAME		Mean (n=81)	Standard deviation (n=81)	CV intraday (%)	CV interday (%)	Trueness (%)
		Certified value	Uncertainty	g/100g fatty acids				
MYR	C14:0	2.29	0.04	2.18	0.63	19.23	28.85	95.00
PAL	C16:0	25.96	0.30	27.76	2.99	7.19	10.78	106.94
PO	C16:1	2.58	0.16	2.12	0.30	10.42	15.63	82.32
STE	C18:0	18.29	0.17	19.70	2.62	8.86	13.29	107.71
OL	C18:1	38.3	0.40	37.57	4.58	8.13	12.20	98.09
LA	C18:2	7.05	0.17	7.01	1.10	10.48	15.72	99.49
ALA	C18:3	0.86	0.14	0.87	0.19	14.71	22.06	101.37

Table S4. Element composition in Cambodian farmed freshwater fish (mg/kg of fresh sample)

Species	Ca	Cu	Fe	K	Mn	Mo	Na	P	Se	Zn
BEC	43.6	0.20	3.62	1581.9	0.09	<LOD	206.1	1019.9	0.17	5.87
CP	3735.8	0.26	4.40	1780.5	1.72	<LOD	344.4	3219.6	0.15	4.57
CC	1900.9	0.46	6.59	2355.4	1.79	<LOD	446.2	2558.4	0.26	15.94
GSH	847.9	0.10	3.19	2041.4	0.37	<LOD	205.2	1620.0	0.15	4.16
IC	2309.2	0.24	5.10	3723.2	2.33	<LOD	488.5	3420.0	0.45	7.80
NT	3611.7	0.34	5.94	2406.0	6.18	0.009	597.4	3231.5	0.34	8.94
PG	8612.0	0.34	4.53	2242.0	5.63	<LOD	351.5	5890.1	0.21	14.78
SB	293.1	0.32	3.39	1761.0	0.24	<LOD	211.4	1276.8	0.19	10.70
SSH	1316.2	0.14	3.05	1840.5	0.89	<LOD	191.0	1800.8	0.09	7.56
WC	82.0	0.15	4.63	2710.2	0.11	<LOD	376.1	1564.2	0.13	7.05
**DORM-4**	**2524.5**	**16.3**	**380.9**	**13593.9**	**3.18**	**0.275**	**14326.2**	**7990.8**	**3.40**	**55.04**
**GBW07603**	**17810.9**	**6.71**	**1208.8**	**9950.2**	**68.1**	**0.292**	**19876.2**	**941.7**	**0.153**	**61.54**
**LOD**	**0**	**0.02**	**0.05**	**0.71**	**0.002**	**0.001**	**0**	**0**	**0.005**	**0.004**
**LOQ**	**0**	**0.06**	**0.17**	**2.39**	**0.009**	**0.006**	**0**	**0**	**0.01**	**0.01**

SSH: *C. striata*; WC: *C. batrachus*; NT: *O. niloticus*; PG: *P. hypophthalmus*; SB: *B. gonionotus*; CC: *C. carpio*; IC: *L. rohita*; GSH: *C. micropeltes;* BEC: P. *larnaudii*; CP: A. *testudineus*; DORM-4 (CRM: fish protein); GBW07603 (CRM: bush branches and leaves); LOD: Limit of Detection; LOQ: Limit of Quantification

Table S5. Fatty acid profile in farmed freshwater fish in Cambodia expressed as a gram of fatty acid per 100 grams of total fatty acids (g/100 g of total fatty acids)

Fatty acids	SSH	WC	SB	NT	CC	IC	GSH	CP	BEC	PG	LOD	LOQ
C10:0	<LOQ	<LOQ	<LOQ	<LOQ	<LOQ	<LOQ	<LOQ	<LOQ	<LOQ	<LOQ	0.3	0.6
C12:0	<LOQ	<LOQ	<LOQ	<LOQ	<LOQ	<LOQ	<LOQ	<LOQ	<LOQ	<LOQ	0.1	0.3
C13:0	<LOQ	<LOQ	<LOQ	<LOQ	<LOQ	<LOQ	<LOQ	<LOQ	<LOQ	<LOQ	0.4	0.8
C14:0	<LOQ	2.7	3.9	3.8	2.3	2.5	3.8	1.1	<LOQ	2.6	0.1	0.3
C16:0	29.9	37.6	41.2	38.4	27.0	30.2	35.8	30.8	35.7	37.6	0.3	0.7
C17:0	<LOQ	2.5	1.2	2.5	1.3	3.4	3.3	<LOQ	<LOQ	1.0	0.1	0.3
C18:0	10.0	10.1	9.4	10.2	7.6	10.4	12.3	9.1	8.0	10.0	0.2	0.5
C20:0	<LOQ	<LOQ	<LOQ	<LOQ	<LOQ	<LOQ	1.1	<LOQ	<LOQ	<LOQ	0.1	0.3
C22:0	<LOQ	<LOQ	<LOQ	<LOQ	<LOQ	<LOQ	<LOQ	<LOQ	<LOQ	<LOQ	0.1	0.3
C24:0	0.5	<LOQ	<LOQ	<LOQ	<LOQ	<LOQ	<LOQ	<LOQ	<LOQ	<LOQ	0.1	0.3
C16:1	1.5	6.4	<LOQ	<LOQ	<LOQ	4.4	8.3	<LOQ	5.6	3.3	0.1	0.3
C17:1	<LOQ	<LOQ	<LOQ	<LOQ	<LOQ	<LOQ	<LOQ	<LOQ	<LOQ	<LOQ	0.4	0.9
C18:1,9t	<LOQ	<LOQ	<LOQ	<LOQ	<LOQ	<LOQ	<LOQ	<LOQ	<LOQ	<LOQ	0.2	0.4
C18:1,9c	20.2	3.1	14.8	3.5	10.3	<LOQ	<LOQ	20.6	24.3	20.3	0.5	1.2
C18:1,11c	<LOQ	<LOQ	<LOQ	<LOQ	<LOQ	<LOQ	<LOQ	<LOQ	<LOQ	<LOQ	0.2	0.4
C22:1	<LOQ	<LOQ	<LOQ	<LOQ	<LOQ	<LOQ	<LOQ	<LOQ	<LOQ	<LOQ	0.2	0.4
C18:2,9c,12c	29.7	15.2	15.7	16.5	22.4	7.3	6.2	29.8	21.9	16.2	0.3	0.6

C18:2,9t,12t	<LOQ	<LOQ	<LOQ	<LOQ	<LOQ	<LOQ	<LOQ	<LOQ	<LOQ	<LOQ	0.2	0.4
C18:2,9c,11t	<LOQ	<LOQ	<LOQ	<LOQ	<LOQ	<LOQ	<LOQ	<LOQ	<LOQ	<LOQ	0.2	0.4
C18:2,9t,11t	<LOQ	<LOQ	<LOQ	<LOQ	<LOQ	<LOQ	<LOQ	<LOQ	<LOQ	<LOQ	0.5	1.2
C18:3 (n-6)	2.3	1.1	<LOQ	1.5	<LOQ	1.3	<LOQ	2.9	0.8	<LOQ	0.1	0.3
C20:2	<LOQ	<LOQ	<LOQ	<LOQ	<LOQ	1.0	<LOQ	<LOQ	<LOQ	<LOQ	0.1	0.3
C20:4	1.5	4.0	2.4	5.2	4.4	8.5	6.7	2.5	1.6	2.8	0.2	0.5
C18:3 (n-3)	1.7	5.2	2.3	3.9	3.1	6.1	4.7	1.9	1.3	2.1	0.2	0.5
C18:4	<LOQ	<LOQ	<LOQ	<LOQ	<LOQ	<LOQ	<LOQ	<LOQ	<LOQ	<LOQ	0.1	0.3
C20:3	<LOQ	<LOQ	<LOQ	<LOQ	<LOQ	<LOQ	<LOQ	<LOQ	<LOQ	<LOQ	0.2	0.5
C20:5	<LOQ	4.4	3.1	<LOQ	4.4	8.5	4.0	<LOQ	<LOQ	<LOQ	0.2	0.5
C22:5	<LOQ	1.9	<LOQ	3.0	2.2	2.9	3.8	<LOQ	<LOQ	1.2	0.2	0.5
C22:6	2.7	5.8	6.0	11.5	14.9	13.6	10.0	1.3	0.9	3.0	0.2	0.5
**SFA**	**40.4**	**52.9**	**55.7**	**55.1**	**38.2**	**46.5**	**56.3**	**41.0**	**43.7**	**51.2**		
**MUFA**	**21.7**	**9.5**	**14.8**	**3.5**	**10.3**	**4.4**	**8.3**	**20.6**	**29.9**	**23.5**		
**PUFA**	**37.9**	**37.6**	**29.5**	**41.4**	**51.5**	**49.1**	**35.4**	**38.4**	**26.4**	**25.3**		
**Total n-6**	**33.5**	**20.3**	**18.1**	**23.1**	**26.8**	**18.0**	**12.9**	**35.2**	**24.2**	**19.0**		
**Total n-3**	**4.4**	**17.3**	**11.4**	**18.3**	**24.6**	**31.1**	**22.5**	**3.2**	**2.2**	**6.3**		
**n-6/n-3**	**7.6**	**1.2**	**1.6**	**1.3**	**1.1**	**0.6**	**0.6**	**11.1**	**11.1**	**3.0**		
**PUFA/SFA**	**0.94**	**0.71**	**0.54**	**0.76**	**1.34**	**1.05**	**0.64**	**0.94**	**0.61**	**0.50**		
**EPA+DHA**	**2.7**	**10.2**	**9.1**	**11.5**	**19.3**	**22.1**	**14.0**	**1.3**	**0.9**	**3.0**		

**AI**	**0.50**	**1.01**	**1.25**	**1.17**	**0.58**	**0.74**	**1.13**	**0.58**	**0.63**	**0.96**
**TI**	**0.94**	**0.73**	**1.03**	**0.75**	**0.39**	**0.40**	**0.64**	**1.05**	**1.24**	**1.20**
**h/H**	**1.20**	**0.91**	**0.66**	**0.95**	**1.76**	**1.43**	**0.90**	**1.12**	**0.72**	**0.63**

LOD: Limit of detection; LOQ : Limit of quantification ; <LOQ: lower than the limit of quantification; EPA: eicosapentaenoic acid; DHA: docosahexaenoic acid; SFA: Saturated fatty acid; MUFA: Monounsaturated fatty acid; PUFA: Polyunsaturated fatty acid; AI: Atherogenicity index; IT: Thrombogenicity index; (h/H); hypocholesterolemic/hypercholesterolemic ratio; SSH: C. striata; WC: C. batrachus; NT: O. niloticus; PG: P. hypophthalmus; SB: B. gonionotus; CC: C. carpio; IC: L. rohita; GSH: C. micropeltes; BEC: P. larnaudii; CP: A. testudineus.

Table S6. Nutritional contribution (%) of farmed freshwater fish in terms of essential elements and EPA and DHA, considering a consumption of 90 g per day.

Nutrients	Adult consumers	Nutritional Contributions (NC %)										Daily Intake Recommendations (mg/day)
		SSH	WC	SB	NT	CC	IC	GSH	CP	BEC	PG	
Ca	Male/Female	11.85	0.74	2.64	32.51	17.11	20.78	7.63	17.11	0.39	77.51	1000[a]
Cu	Male/Female	1.43	1.52	3.25	3.44	4.57	2.42	1.00	4.57	1.98	3.38	0.90[a]
Fe	Male	3.43	5.20	3.82	6.69	7.42	5.74	3.59	7.42	4.07	5.10	8.00[a]
	Female	1.52	2.31	1.70	2.97	3.30	2.55	1.60	3.30	1.81	2.27	18.00[a]
Mn	Male	3.49	0.43	0.93	24.20	7.02	9.14	1.45	7.02	0.35	22.01	2.30[a]
	Female	4.46	0.55	1.18	30.92	8.97	11.67	1.85	8.97	0.45	28.13	1.80[a]
Mo	Male/Female	0.04	0.06	0.06	1.74	0.16	0.06	0.04	0.16	0.08	0.06	0.045[a]
P	Male/Female	23.15	20.11	16.42	41.55	32.89	43.97	20.83	32.89	13.11	75.73	700[a]
K	Male	4.87	7.17	4.66	6.37	6.24	9.86	5.40	6.24	4.19	5.93	3400[a]
	Female	6.37	9.38	6.10	8.33	8.15	12.89	7.07	8.15	5.48	7.76	2600[a]
Se	Male/Female	14.37	21.47	31.34	55.42	42.95	73.64	25.22	42.95	28.16	34.02	0.055[a]
Na	Male	1.15	2.26	1.27	3.58	2.68	2.93	1.23	2.68	1.24	2.11	1500[a]
Zn	Male	6.19	5.77	8.76	7.32	13.04	6.38	3.40	13.04	4.80	12.09	11.00[a]
	Female	8.51	7.93	12.04	10.06	17.93	8.78	4.68	17.93	6.60	16.63	8.00[a]
EPA+DHA	Male/Female	18.81	169.55	140.08	43.69	109.14	64.13	56.76	109.14	28.03	38.80	500[b]

EPA:Eicosapentaenoic acid; DHA: Docosahexaenoic acid; NC: Nutritional contribution; SSH: C. striata; WC: C. batrachus; NT: O. niloticus; PG: P. hypophthalmus ; SB: B. gonionotus; CC: C. carpio; IC: L. rohita; GSH: C. micropeltes; BEC: P. larnaudii; CP: A. testudineus; [a](Food and Nutrition Board, 2019); [b](EFSA, 2010).
